# Prevalence of Infectious Hypodermal and Hematopoietic Necrosis Virus (IHHNV) in Farmed *Procambarus clarkii* of the Middle and Lower Reaches of the Yangtze River in China

**DOI:** 10.3390/pathogens12081038

**Published:** 2023-08-14

**Authors:** Feng Xu, Yongwei Wei, Jianfei Lu, Jiong Chen

**Affiliations:** 1 Central Laboratory of the Medical Research Center, The First Affiliated Hospital of Ningbo University, Ningbo 315211, China; 2 State Key Laboratory for Managing Biotic and Chemical Threats to the Quality and Safety of Agro-products, Ningbo University, Ningbo 315211, China; 3 Laboratory of Biochemistry and Molecular Biology, School of Marine Sciences, Ningbo University, Ningbo 315211, China; 4 Key Laboratory of Aquacultral Biotechnology Ministry of Education, Ningbo University, Ningbo 315211, China

**Keywords:** IHHNV, *Procambarus clarkii*, prevalence, capsid protein gene

## Abstract

*Procambarus clarkii* is an important economic aquaculture species worldwide. Infectious hypodermal and hematopoietic necrosis virus (IHHNV) infects numerous crustacean hosts, including *P. clarkii*. However, there have been few reports on the prevalence of IHHNV in *P. clarkii*. In this study, 200 farmed *P. clarkii* were collected from Anhui, Jiangsu, Zhejiang, Hunan, Hubei, and Sichuan provinces in China. PCR detection was employed per the protocol by the World Organization for Animal Health (WOAH) to identify and detect the presence of IHHNV. The positive rate of IHHNV in different provinces ranged from 16.7 to 56.7%, and the overall IHHNV-positive rate was 38.5%. IHHNV strains isolated in this study related closely to infectious IHHNV and split into two major distinct branches. Besides, the IHHNV strains shared a high homology (93.4–99.4%). These findings suggest that a high prevalence of IHHNV was established in farmed *P. clarkii* in the middle and lower reaches of the Yangtze River.

## 1. Introduction

Infectious hypodermal and hematopoietic necrosis virus (IHHNV) is an economically crucial pathogen that results in a high mortality rate in aquaculture [1,2]. IHHNV belongs to the genus *Brevidensovirus* of the Parvoviridae family, also named Decapod penstyldensovirus 1 (PstDV1) or Penaeus sytlirostris brevidensovirus (PstDNV) [3]. The genome of IHHNV is 3.9 kb DNA in length, encoding the nonstructural protein 1 (NS-1), non-structural protein 2 (NS-2), and capsid protein (CP), respectively [4]. Since its initial report in *Penaeus stylirostris* in Hawaii during the early 1980s, IHHNV has caused significant economic damage around the world. Consequently, it has been listed as one of the notifiable crustacean pathogens by the World Organization for Animal Health (WOAH) [1]. To date, IHHNV has been detected in several aquatic economic species, including penaeid shrimp, Cambarus, crab, and bivalve shellfish [5,6,7,8,9].

IHHNV was initially identified in China in 2001. Yang et al. detected IHHNV in penaeid shrimp and *Hemigrapsus penicillatus* samples in Hainan Province. The observed prevalence of IHHNV infection was 51.5% among penaeid shrimp and 8.3% among *H. penicillatus* samples [10]. In recent years, IHHNV has been detected in many provinces in China, including Zhejiang, Jiangsu, Shanghai, Anhui, Shandong, Fujian, Hainan, Guangxi, and Guangdong [2]. IHHNV infection in *P. stylirostris* and *Macrobrachium rosenbergii* juveniles causes a high mortality rate [1,11,12]. IHHNV causes chronic infections known as runt-deformity syndrome (RSD) in *Litopenaeus vannamei* and *Penaeus monodon* due to its effect on the reduction of growth performance [13]. However, crabs and bivalve shellfish primarily act as carriers of IHHNV, showing no discernible symptoms of infection [2].

*Procambarus clarkii,* a member of crustacea, decapoda, and cambaridae, has become a momentous economic aquaculture species in China [14,15]. Recently, IHHNV has been detected in cultured *P. clarkii*. The mortality of *P. clarkii* artificially infected with IHHNV is high [16]. However, whether IHHNV affects farmed *P. clarkii* remains unknown. In this study, we investigated IHHNV’s prevalence in farmed *P. clarkii* in the middle and lower reaches of the Yangtze River of China. CP genes of the detected IHHNV were sequenced and analyzed for comparisons of genetic variations with other IHHNV strains. Additionally, we analyzed the histological section of IHHNV-positive *P. clarkii*.

## 2. Materials and Methods

### 2.1. Samples

**Figure 1:**
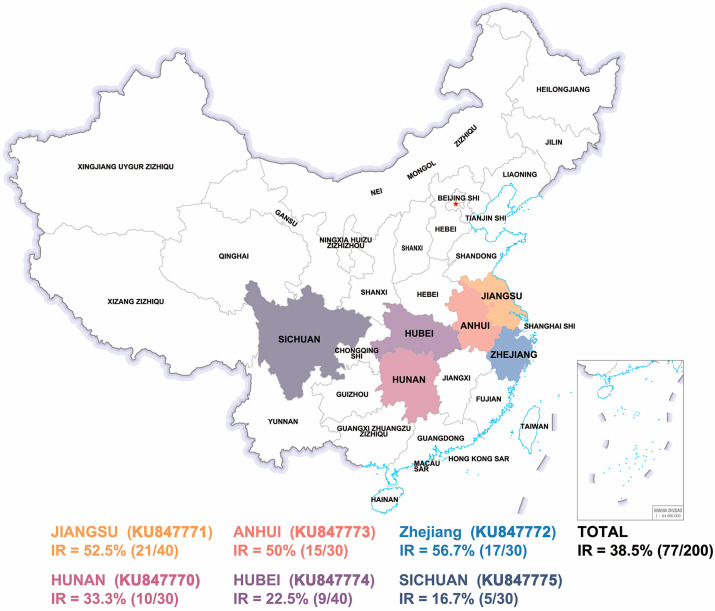
Provinces of China where IHHNV surveillance described in this report was conducted. The colored text indicates the province name and IHHNV-positive rate (IR).

Two-hundred farmed healthy *P. clarkii* were collected from the middle and lower reaches of the Yangtze River region (Changjiang River) in China. Sampling sites were distributed in the Anhui (30), Jiangsu (40), Zhejiang (30), Hunan (30), Hubei (40), and Sichuan (30) provinces labeled in Figure 1. All samples from each region were assembled in one week, while the samples from the same region were collected in one day. The collected samples of *P. clarkii* have an average body length of 10 ± 1 cm and an average body weight of 25 ± 5 g. The samples were transported to the laboratory at a low temperature and further experiments were conducted immediately. The experiments were approved by the Committee on Animal Care and Use and the Committee on the Ethics of Animal Experiments of Ningbo University.

### 2.2. Identification of IHHNV

DNA was extracted from gills and pleopod from each *P. clarkii* by using a DNA Extraction Kit (TaKaRa, Japan) following the manufacturer’s protocol. Briefly, 20 mg of tissues were homogenized by cutting and grinding. The homogenate was then incubated with 180 μL of buffer GL, 20 μL of proteinase K, and 10 μL of RNase A at 56 °C. After pyrolysis, impurities were removed by centrifugation at 12,000 rpm for 2 min. Subsequently, 200 μL buffer GB and 100% ethanol were added to the supernatant. The resulting solution was transferred to a spin column and centrifuged at 12,000 rpm for 2 min. Wash steps with buffer WA and buffer WB were performed consecutively. Finally, elution buffer (50 μL) was added and the mixture was centrifuged at 12,000 rpm for 2 min to elute DNA. The purity of the extracted DNA was assessed using a Nano Photometer N60 Spectrophotometer (Implen, Germany). 

Polymerase chain reaction (PCR) detection was carried out according to the WOAH suggested protocol using the primers of 389F: 5′-CGGAACACAACCCGACTTTA-3′ and 389R: 5′-GGCCAAGACCAAAATACGAA-3′. PCR amplification was performed at 94 °C, 5 min, followed by 32 cycles at 95 °C for 30 s, 56 °C for 30 s, and 72 °C for 30 s followed by a 72 °C extension for 10 min. The PCR products were mixed with a DNA loading dye and loaded onto 2% agarose gels. Electrophoresis was performed at 120 V for 20 min. Subsequently, the DNA fragments within the gel were visualized using DNA-specific dyes, such as ethidium bromide, under UV light.

### 2.3. Acquisition of IHHNV CP Gene

To perform phylogenetic analysis, three IHHNV-positive samples were randomly selected from each region for CP gene amplification. The primers were IHHNV-CP-F: (5′-TCCAAGAATACGAAAAGGAAATC-3′) and IHHNV-CP-R (5′-AGAGGGTAGGTATAGATAGTAATAGA-3′). PCR amplification was performed at 94 °C, 5 min, followed by 32 cycles at 95 °C for 30 s, 56 °C for 30 s, and 72 °for C 60 s followed by a 72 °C extension for 10 min. The products were detected by using 1% agarose gel. In addition, the positive PCR products were cloned to the pMD-19T vector (TaKaRa, Japan), and samples were sent to the Sangon Biotech (Shanghai, China) for sequencing.

### 2.4. Bioinformatics Analysis of CP Genes

To classify IHHNV of different regions, the phylogenetic relationship was obtained by comparing the nucleotide sequences with those of other IHHNV capsid proteins in the GenBank database. The ClustalW 2.0 program was used for sequence alignment (http://clustalw.ddbj.nig.ac.jp/ (accessed on 15 September 2022)). Phylogenetic and molecular evolution were analyzed by the Neighbor-Joining method through 1000 bootstrap replicates using the MEGA 7.0 software (https://academic.oup.com/mbe/article/33/7/1870/2579089?login=false (accessed on 15 September 2022)).

## 3. Results

Thirty *P. clarkii* samples collected from Zhejiang farms were detected by PCR according to the WOAH-suggested protocol. The results showed that 17 out of 30 samples were IHHNV-positive, a positive rate of 56.7% (Figure 1). Furthermore, we expanded our investigation to cover the middle and lower reaches of the Yangtze River where *P. clarkii* is mainly farmed in China. Aggregate 170 samples were collected in the Anhui, Jiangsu, Hunan, Hubei, and Sichuan provinces, and the sampling map is shown in Figure 1. Samples were detected by PCR assay according to the method described above. The PCR analysis revealed that the IHHNV-positive rate was 21/40 (52.5%) in Jiangsu, 15/30 (50%) in Anhui, 10/30 (33.3%) in Hunan, 9/40 (22.5%) in Hubei, and 5/30 (16.7%) in Sichuan. Overall, the positive rate of IHHNV in *P. clarkii* of all the samples was over 38.5%.

**Figure 2:**
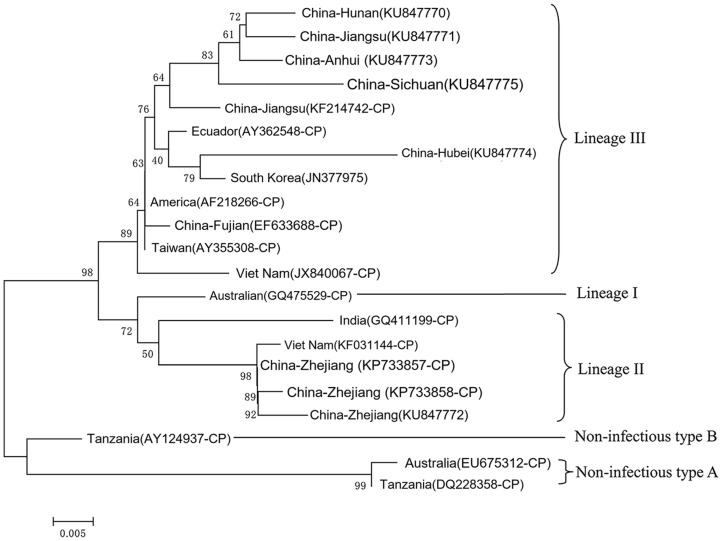
Phylogenetic analysis of IHHNV by CP gene. IHHNV isolated in this report were named using location and GenBank ID.

To classify the IHHNV identified above, the complete CP gene sequence was cloned and sequenced. The CP gene sequence was submitted to NCBI with the accession number: KU847770 (Hunan), KU847771 (Jiangsu), KU847772 (Zhejiang), KU847773 (Anhui), KU847774 (Hubei), and KU847775 (Sichuan). IHHNV was divided into five genotypes based on genome sequences in GenBank, including infectious IHHNV strains (lineages I-III) and non-infectious IHHNV type A/B (Tang et al., 2003). In the phylogenetic analysis based on the CP gene of IHHNV, the IHHNV strains isolated in this study were discovered to be related closely to infectious IHHNV (Figure 2). Besides, the phylogenetic tree of IHHNV from *P. clarkii* was split into two major distinct branches. Lineage I is composed exclusively by strain GQ475529. The strains of Zhejiang (KU847772), GQ411199, KF031144, KP733857, and KP733858 formed a cluster with lineage II. In addition, IHHNV from the Anhui, Jiangsu, Hunan, Hubei, and Sichuan strains formed a cluster with viruses KF214742, AY362548, JN377975, AF218266, EF633688, AY355308, and JX840067 in lineage III. These data indicate that *P. clarkii* is a carrier of different IHHNV lineages. Interestingly, the Zhejiang strain belonged to lineage II alone, while IHHNV from Anhui, Jiangsu, Hunan, Hubei, and Sichuan were in lineage I. 

To evaluate the genetic relationships among those IHHNV strains, CP gene sequences isolated in this study were aligned with five genotypes of IHHNV. The results demonstrated that the IHHNV strains isolated in the middle and lower reaches of the Yangtze River shared a high homology (93.4–99.4%) (Table 1). The highest homology was found in KU847770 (Hunan) and KU847771 (Jiangsu), and the lowest homology was found in KU847772 (Zhejiang) and KU847773 (Anhui). IHHNV from Zhejiang, Hunan, Jiangsu, Anhui, and Sichuan shared the highest similarity with AF218266, while IHHNV from Hubei shared the highest similarity with JN377975. Additionally, IHHNV strains isolated in the middle and lower reaches of Yangtze River shared the lowest similarity with EU675312, and the level of identity was 86.2–87.0%. Compared with infections IHHNV strains, IHHNV isolated in the middle and lower reaches of the Yangtze River shared the lowest similarity with non-infection IHHNV strains (up to 86.2%). 

Despite the genetic variation rate of the parvovirus genome being low (up to 4%), the genome of IHHNV exhibited uniquely high variation rates (14%) [17,18]. The genetic variation rate of the IHHNV CP gene was up to 13.2% [19]. In this study, the CP gene was also in accord with previous results showing that the variation rate slightly increased (up to 13.8%). Our results verified again that although IHHNV is a DNA virus, the mutation rate of the virus is as high as an RNA virus [18].

## 4. Discussion

To investigate the prevalence of IHHNV in farmed *P. clarkii*, samples were collected and detected from five provinces in the middle and lower reaches of the Yangtze River, China. Unexpectedly, IHHNV was detected in all the tested provinces. The positive rate of IHHNV in different provinces ranged from 16.7% to 56.7%, and the overall IHHNV-positive rate was 38.5%. To sum up, the high prevalence of IHHNV indicates that IHHNV is widespread in farmed *P. clarkii* populations.

In addition to *P. clarkii*, more than 30 animals have been reported to be infected by IHHNV or just as natural carriers of IHHNV [2]. The commonly farmed penaeid shrimp species all have been infected by IHHNV, including *Penaeus vanmamei*, *P. stylirostris*, *Penaeus occidentalis*, *P. monodon*, etc. [20]. In some provinces of China, the IHHNV-positive rate in cultured *P. vanmamei* exceeded 90% [2]. The positive rate of IHHNV in *Mytilus edulis* along the coast of China was up to 90% [9]. Besides, a 15–50% IHHNV-positive rate was found in bivalve shellfish and snails, including *Mactra chinensis*, *Tegillarca granosa*, *Ruditapes philippinarum*, *Sinonovacula constricta*, *Meretrix meretrix*, *Mactra veneriformis*, *Margarya melanioides*, and *Cipangopaludina cahayensis* [21]. There has been little research conducted on whether *P. clarkii* can be infected with IHHNV. The first reported study of *P. clarkii* infected with IHHNV was in 2017; they detected IHHNV in farmed *P. clarkii* and found that *P. clarkii* could be artificially infected by IHHNV [16]. In the present study, we detected IHHNV in farmed *P. clarkii* in the middle and lower reaches of the Yangtze River region, and the IHHNV-positive rate of *P. clarkii* ranged from 16.7 to 56.7%. These data illustrated that *P. clarkii* is a common host of IHHNV.

IHHNV has a variety of hosts, especially those in crustaceans. Most of the penaeid shrimp species can be naturally or experimentally infected with IHHNV [22]. This virus is prevalent among penaeid shrimp and results in a significant decrease in shrimp production [2]. When it was first discovered, it was responsible for the high mortality rate of penaeid shrimp [1]. However, few high mortality rates caused by IHHNV have been reported in recent years [2]. IHHNV causes chronic infections known as RSD [23] in *L. vannamei* [13]. *P. clarkii* also can be naturally and experimentally infected by IHHNV. Artificial infection of *P. clarkii* with IHHNV causes enlarged nuclei symptoms in gills and can cause high mortality [16]. Our study found that healthy *P. clarkii* was IHHNV-positive. However, the impact of IHHNV infection on farmed *P. clarkii* is still unknown, and further follow-up research is needed. These results indicate that the farmed *P. clarkii* in the Yangtze River basin of China were natural carriers of IHHNV.

Phylogenetic analysis showed that IHHNV from Zhejiang belonged to lineage III alone, while IHHNV from Anhui, Jiangsu, Hunan, Hubei, and Sichuan formed a distinct cluster and belonged to lineage II, suggesting a common origin for Anhui, Jiangsu, Hunan, Hubei, and Sichuan strains. Jiangsu, Hunan, Hubei, and Sichuan are geographically closer together, while Zhejiang is relatively far away from them. Therefore, it is reasonable to speculate that IHHNV transmission may have more frequent exchanges between Anhui, Jiangsu, Hunan, Hubei, and Sichuan. The spread of IHHNV in different provinces may be the result of seedling and mature body transport. The detailed transmission mechanism of IHHNV in Anhui, Jiangsu, Hunan, Hubei, Sichuan, and Zhejiang is unknown and needs further study. Understanding this process will help elucidate how IHHNV emerges, spreads, and breaks out.

IHHNV has caused serious economic losses in the cultivation of crustaceans, and the potential risks associated with its spread are of concern. However, the precise mechanisms underlying infection, transmission, and host–virus interactions at the cellular and molecular levels remain poorly understood. Numerous studies have highlighted a high prevalence of this virus, warranting further investigation into the consequences for crustacean populations. Particularly noteworthy is the asymptomatic carrier status exhibited by multiple crustacean species, which greatly amplifies the risk of inter-species transmission. Consequently, future work should prioritize comprehensive epidemiological investigations of IHHNV and the elucidation of its molecular infection mechanisms.

In conclusion, our data demonstrated the high prevalence of IHHNV in farmed *P. clarkii* in the middle and lower reaches of the Yangtze River. In particular, IHHNV in *P. clarkii* shares high homology with the infectious IHHNV strains. Therefore, IHHNV is an important pathogen posing a serious threat to the shrimp farming industry in China.

## Figures and Tables

**Table 1:** Nucleotide percent similarity and divergence among CP gene of IHHNV.

		1	2	3	4	5	6	7	8	9	10	11	12	13	14	15	16	17	18	19	20	21
GQ475529	1		93.7	95.4	95.1	95.1	94.5	94.3	94.5	94.3	94.3	94.9	95.3	94.2	95.2	95.6	95.4	95.5	95.2	87.2	87.1	91.4
GQ411199	2	6.6		92.9	93.1	92.8	92.7	92.6	92.8	92.6	92.5	93.0	93.3	92.2	93.2	93.6	93.6	93.7	93.2	86.6	86.5	90.3
KF031144	3	4.8	7.5		99.7	99.7	99.2	94.2	94.4	94.0	94.4	94.6	94.9	94.3	94.8	95.3	95.1	95.2	94.8	97.3	87.2	91.3
KP733857	4	5.1	7.2	0.3		99.6	99.5	94.1	94.3	93.9	94.3	94.5	94.8	94.2	94.7	95.2	94.9	95.1	94.7	87.2	87.1	91.2
KP733858	5	5.1	7.6	0.3	0.4		99.1	94.1	94.3	93.9	94.3	94.5	94.8	94.2	94.7	95.2	94.9	95.1	94.7	97.3	87.2	91.3
	6	5.7	7.7	0.8	0.5	0.9		93.6	93.8	93.4	93.8	94.0	94.3	93.7	94.2	94.6	94.4	94.5	94.2	87.1	87.0	90.8
	7	5.9	7.8	6.0	6.1	6.1	6.7		99.4	99.2	98.6	98.6	98.5	97.2	98.2	98.6	98.4	98.5	98.2	86.6	86.5	90.6
KU847771	8	5.7	7.5	5.8	5.9	5.9	6.5	0.6		99.2	98.8	98.6	98.7	97.4	98.4	98.8	98.6	98.7	98.4	86.8	86.7	90.8
KU847773	9	5.9	7.8	6.2	6.3	6.3	6.9	0.8	0.8		98.6	98.4	98.3	97.0	98.0	98.4	98.2	98.3	98.0	86.5	86.4	90.4
KU847775	10	5.9	7.9	5.8	5.9	5.9	6.5	1.4	1.2	1.4		98.2	98.3	97.2	98.0	98.4	98.2	98.3	98.0	86.4	86.3	90.4
KF214742	11	5.2	7.3	5.6	5.7	5.7	6.2	1.4	1.4	1.6	1.8		99.1	97.8	98.8	99.2	99.0	99.1	98.8	86.8	86.7	90.6
AY362548	12	4.9	7.0	5.2	5.4	5.4	5.9	1.5	1.3	1.7	1.7	0.9		98.5	99.5	99.5	99.3	99.4	99.0	87.1	87.0	90.9
KU847774	13	6.0	8.2	5.9	6.0	6.0	6.6	2.9	2.7	3.1	2.9	2.3	1.5		98.8	98.4	98.2	98.3	97.9	86.3	86.2	90.1
JN377975	14	5.0	7.1	5.4	5.5	5.5	6.0	1.8	1.6	2.1	2.1	1.2	0.5	1.2		99.4	99.2	99.3	98.9	87.1	87.0	90.8
AF218266	15	4.6	6.7	4.9	5.0	5.0	5.6	1.4	1.2	1.6	1.6	0.8	0.5	1.6	0.6		99.8	99.9	99.5	87.6	87.5	91.2
EF633688	16	4.8	6.7	5.1	5.2	5.2	5.8	1.6	1.4	1.8	1.8	1.0	0.7	1.8	0.8	0.2		99.9	99.3	97.4	87.3	91.0
AY355308	17	4.7	6.5	5.0	5.1	5.1	5.7	1.5	1.3	1.7	1.7	0.9	0.6	1.7	0.7	0.1	0.1		99.4	87.5	87.4	91.1
JX840067	18	5.0	7.1	5.4	5.5	5.5	6.0	1.8	1.6	2.1	2.1	1.2	1.0	2.2	1.1	0.5	0.7	0.6		87.1	87.0	90.7
DQ228358	19	14.1	14.8	14.0	14.1	14.0	14.2	14.9	14.6	15.0	15.1	14.6	14.2	15.2	14.2	13.6	13.9	13.7	14.2		99.7	89.1
EU675312	20	14.3	15.0	14.1	14.2	14.1	14.4	15.0	14.7	15.1	15.2	14.7	14.4	15.4	14.4	13.7	14.0	13.9	14.4	0.3		89.0
AY124937	21	9.1	10.4	9.2	9.4	9.2	9.8	10.1	9.8	10.3	10.3	10.1	9.7	10.7	9.8	9.4	9.6	9.5	9.9	11.8	12.0	

## Data Availability

The data presented in this study are available on request from the corresponding author.
